# Envelope Determinants of Equine Lentiviral Vaccine Protection

**DOI:** 10.1371/journal.pone.0066093

**Published:** 2013-06-13

**Authors:** Jodi K. Craigo, Corin Ezzelarab, Sheila J. Cook, Liu Chong, David Horohov, Charles J. Issel, Ronald C. Montelaro

**Affiliations:** 1 Center for Vaccine Research, University of Pittsburgh, Pittsburgh, Pennsylvania, United States of America; 2 Department of Microbiology and Molecular Genetics, University of Pittsburgh, Pittsburgh, Pennsylvania, United States of America; 3 Department of Veterinary Science, Gluck Equine Research Center, University of Kentucky, Lexington, Kentucky, United States of America; Center for Biologics Evaluation and Research, United States of America

## Abstract

Lentiviral envelope (Env) antigenic variation and associated immune evasion present major obstacles to vaccine development. The concept that Env is a critical determinant for vaccine efficacy is well accepted, however defined correlates of protection associated with Env variation have yet to be determined. We reported an attenuated equine infectious anemia virus (EIAV) vaccine study that directly examined the effect of lentiviral Env sequence variation on vaccine efficacy. The study identified a significant, inverse, linear correlation between vaccine efficacy and increasing divergence of the challenge virus Env gp90 protein compared to the vaccine virus gp90. The report demonstrated approximately 100% protection of immunized ponies from disease after challenge by virus with a homologous gp90 (EV0), and roughly 40% protection against challenge by virus (EV13) with a gp90 13% divergent from the vaccine strain. In the current study we examine whether the protection observed when challenging with the EV0 strain could be conferred to animals via chimeric challenge viruses between the EV0 and EV13 strains, allowing for mapping of protection to specific Env sequences. Viruses containing the EV13 proviral backbone and selected domains of the EV0 gp90 were constructed and *in vitro* and *in vivo* infectivity examined. Vaccine efficacy studies indicated that homology between the vaccine strain gp90 and the N-terminus of the challenge strain gp90 was capable of inducing immunity that resulted in significantly lower levels of post-challenge virus and significantly delayed the onset of disease. However, a homologous N-terminal region alone inserted in the EV13 backbone could not impart the 100% protection observed with the EV0 strain. Data presented here denote the complicated and potentially contradictory relationship between *in vitro* virulence and *in vivo* pathogenicity. The study highlights the importance of structural conformation for immunogens and emphasizes the need for antibody binding, not neutralizing, assays that correlate with vaccine protection.

## Introduction

The science of preventing infectious diseases is an advancing, ever-evolving discipline re-invigorated continuously by the challenge to overcome persistent infections as well as the emergence of new acute epidemics. While the field of vaccinology has developed new, cutting-edge techniques, utilizing very traditional approaches to vaccine development can still demonstrate important lessons. One such vaccine model is the live-attenuated (attenuated) vaccine. Attenuated virus vaccines are under evaluation for a variety of emerging viral maladies while also currently used for the prevention of infectious diseases such as influenza, chicken pox, and yellow fever, and have effectively controlled substantial viral outbreaks such as smallpox, polio, and measles epidemics [1]–[3]. However, the use of an attenuated human immunodeficiency virus (HIV) vaccine has been controversial due to obvious concerns surrounding vaccine safety [4]–[12]. Regardless of the low potential for public or commercial use of an attenuated vaccine for HIV, the attenuated model itself is, to date, one of the best measures of both potential vaccine efficacy and correlates of protection, and remains an asset to the field of study.

The development of vaccines to HIV-1 has relied substantially on the use of animal lentivirus models to evaluate the efficacy of various vaccine strategies. EIAV, a macrophage-tropic lentivirus, produces a persistent infection in horses and a chronic disseminated disease of worldwide importance in veterinary medicine (reviewed in Craigo and Montelaro, 2008 and Montelaro, Ball, et al. 1993). The virus infection, transmitted via blood-feeding insects or iatrogenic sources (i.e. contaminated syringe needles), occurs in three stages: acute, chronic, and inapparent. EIA is characterized during its acute and chronic stages by well-defined episodes of clinical disease triggered by waves of viremia and distinguished by fever, anemia, thrombocytopenia, edema, and various wasting signs. By 8–12 months post-infection horses typically progress to life-long inapparent carriers, but maintain varying steady state levels of viral replication in monocyte-rich tissue reservoirs [13]–[16]. Stress or immune suppression of EIAV inapparent carriers can induce an increase in viral replication and potentially a recrudescence of disease [17]–[19]. Among virulent lentiviruses, however, EIAV is unique in that despite aggressive virus replication and associated rapid antigenic variation, greater than 90% of infected animals progress from a chronic disease state to an inapparent carrier stage. This progression to an inapparent stage of disease is achieved by a strict immunologic control over virus replication [13], [16]. The EIAV system therefore serves as a uniquely dynamic model for the natural immunologic control of lentiviral replication and disease. Thus, the EIAV model provides a novel and useful lentiviral system for identifying immune correlates of protection and ascertaining the potential for developing effective prophylactic lentivirus vaccines.

Over the past 20 years we have evaluated a number of experimental EIAV vaccines [20]–[28]. Results of these vaccine trials demonstrate a noteworthy breadth of efficacy, ranging from protection from detectable infection and/or disease to severe enhancement of EIAV replication and disease. The most recent of this vaccine work has been an ongoing series of studies focusing on an attenuated EIAV proviral vaccine containing a non-functional viral *S2* accessory gene (EIAV_D9_) [24]–[27]. Results of the initial studies confirmed that the EIAV system mirrors other animal lentivirus vaccine models that have consistently identified attenuated vaccines, as producing the highest level of vaccine protection, typically against homologous virus challenge where EIAV_D9_ confers 100% protection [24]–[27], [29]–[32].

Recent published reports of this attenuated EIAV vaccine system detail the specific effects of Env sequence variation on vaccine protection and associated correlations with protection [27], [28], [33], [34]. We identified for the first time a significant, inverse, linear correlation between vaccine efficacy and increasing divergence of the challenge virus Env surface gp90 protein compared to the vaccine virus gp90 protein. The vaccine study demonstrated approximately 100% protection of immunized horses from disease after challenge by virus with a homologous gp90 (EV0), but less than 50% protection against challenge by virus with a gp90 that was 13% (EV13) divergent from the vaccine strain. Immune analysis of potential correlates of protection between the three challenge groups revealed minor associations, but were not definitive. Most recently we demonstrated that the attenuated vaccine strain progressively evolved during the six-month pre-challenge period and that the observed protection from disease was significantly associated with divergence from the original vaccine strain, not the overall diversity present on the day of challenge (DOC) [28].

Attempts to map immune protection to specific domains of the Env gp90 gene [27] through analysis of humoral and cellular immune responses failed to identify an immune correlate of protection with any level of statistical significance. Here we describe a study to evaluate directly the effect of variant Env gp90 domains on vaccine efficacy in a very controlled and detailed manner by developing new challenge strains with chimeric gp90 sequences. The constructed chimeric proviral EIAV challenge viruses were based on the Env gp90 sequences from the two extremes of EIAV_D9_ vaccine protective efficacy from the 2007, 2010 studies: 100% protection (EV0) vs 40% protection (EV13) [27], [28]. The N-terminus and C-terminus of the EV0 and EV13 were exchanged and two chimeric proviral strains evaluated for pathogenesis and against vaccine protection induced by the EIAV_D9_ attenuated vaccine. Results from both the virulence study and EIAV_D9_ vaccine trial indicate that recombinant chimeric virus strains have a complex nature *in vitro* and *in vivo*.

## Results

### Construction and *in vitro* Testing of Chimeric Proviral EIAV Strains

**Figure 1:**
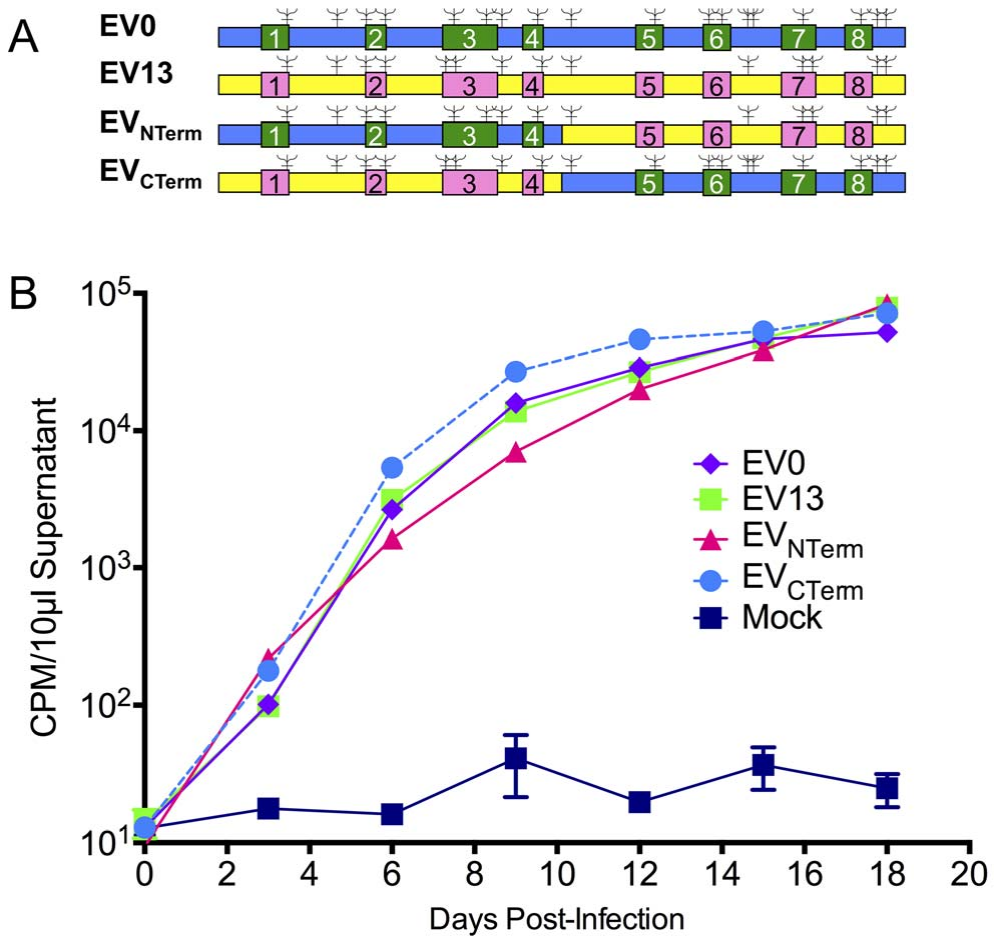
Chimeric proviral strains replicate in vitro in equine cells similar to parental strains. (**A**) Schematic illustration of the parental EIAV strains, EV0 and EV13, and the resulting chimeric strains, EV_NTerm_ and EV_CTerm_. Boxed sites signify the variable regions (numbered 1–8) of the EIAV genome. Blue/green represents EV0-specific genome; Yellow/pink represents EV13-specific genome; = predicted N-linked glycosylation sites. (**B**) Replication kinetics of parental and chimeric strains is plotted as RT activity (CPM/10 µl supernatant) versus days post-infection. Infections were set up with equivalent MOIs (0.1) of parental and chimeric viruses. Supernatants from infected FEK cells and mock-infected cells were collected every three days and assayed for RT activity.

To examine regional-specific genomic affects of the Env gp90 on viral virulence and vaccine efficacy, two chimeric Env gp90 EIAV strains were constructed. Virulent challenge strains from a previous EIAV vaccine study on the affects of Env variation on vaccine efficacy [27], [28] were utilized as parental strains. The two strains, termed EV0 and EV13, were virulent challenge strains that were 13% divergent from each other in their particular gp90 envelope sequences and 0% or 13% divergent, respectively, from the attenuated vaccine strain gp90. Hence, the gp90 sequence of EV0 was homologous to that of the attenuated EIAV_D9_ vaccine strain and animals vaccinated with EIAV_D9_ were protected against challenge with EV0 unlike the animals challenged with EV13 where protection from EIA disease was reduced to approximately 40%. The goal of the chimeric constructs was to confer the vaccine protection phenotype of the EV0 strain into the EV13 backbone by means of the N-terminal or C-terminal (or both) regions of the surface protein. To construct these chimeric strains, the gp90 regions of EV0 and EV13 were both split in the highly conserved genomic region between the fourth and fifth variable domains of the gene (Figure 1A). The corresponding N-terminus and C-terminus of the reciprocal gp90 genes were cloned into the genome of the respective proviral backbones. The resultant chimeric strains, termed EV_NTerm_ and EV_CTerm_, were sequenced to verify the integrity of the viral genome. Viral stocks were generated and tittered. Viral replication kinetics of the two chimeric strains were compared to that of the parental strains by infection of fetal equine kidney (FEK) cells (Figure 1B). Reverse transcriptase activity of the infected-cell supernatants indicated that *in vitro* replication of the two chimeric proviral strains was almost identical to that of their parental stains.

### *In vivo* Virulence and Pathogenesis of Chimeric Proviral EIAV Strains, EV_NTerm_ and EV_CTerm_

**Figure 2:**
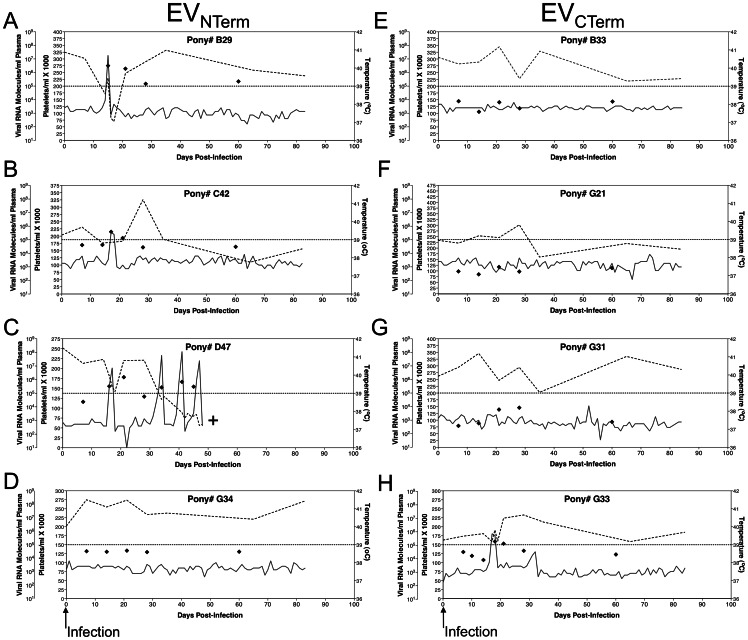
Clinical and virological profiles of chimeric strain pony infections. The profiles depicted in **A–H** display the clinical and virological outcomes observed in EIAV chimeric strain viral infections with EV_NTerm_ (**A–D**) and EV_CTerm_ (**E–H**). After inoculation (0 DPI, ↑ Infection) of EIAV-naïve ponies with 10^3^ TCID_50_ I.V. injections with the respective viral strains their rectal temperatures (**–**, right Y axis) and platelet counts (–-, 1st left Y axis) were followed daily for approximately 90 days (X-axis). Quantification of the plasma virus loads (⧫, 2nd left Y axis) on viral RNA extracted from plasma at periodic time points were performed throughout the acute infections and during potential fever episodes of the chronic stage stages as well. Febrile episodes are defined by achieving a combination of two-three features such as: rectal temperature above 39°C in conjunction with thrombocytopenia (platelet decrease of ≥70,000/µl of whole blood), EIAV viral load ≥10^5^ as well as other clinical signs of EIA. **+**, Animal euthanized due to severe disease.

**Figure 3:**
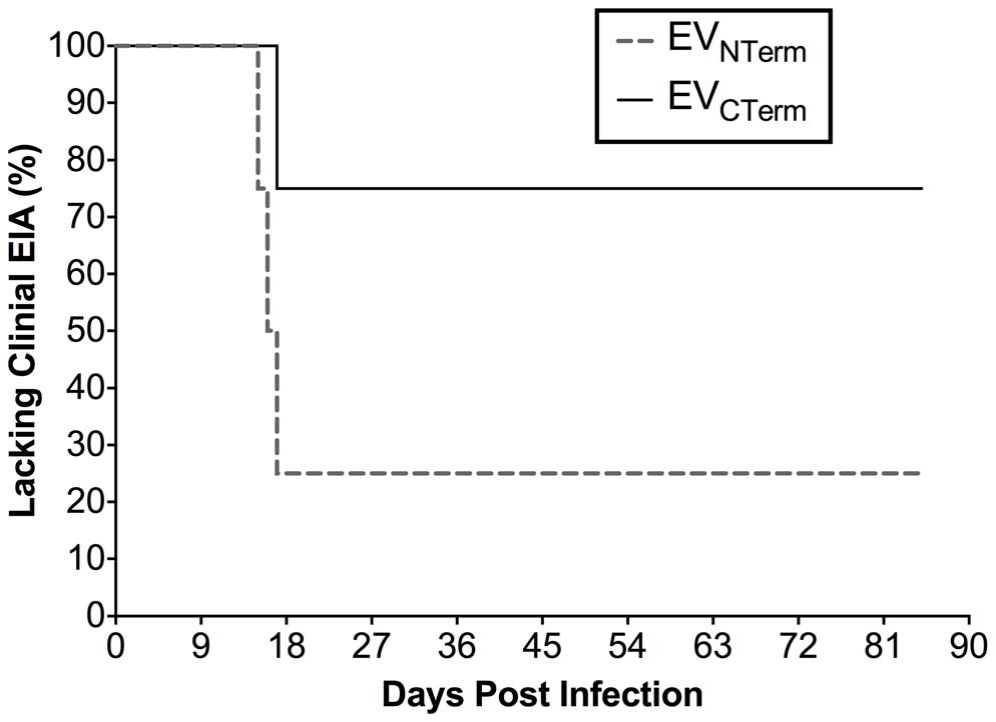
Chimeric strain infections did not result consistently in clinical disease. The percentage of animals within each trial group, EV_NTerm_ and EV_CTerm_ that did not develop clinical EIA was plotted as a function of days post-infection.

The virulence of the chimeric EIAV strains EV_NTerm_ and EV_CTerm_, and their ability to cause disease in equids, was evaluated utilizing our standard *in vivo* infection model of EIAV infection and disease [14], [18], [19], [22], [24]–[27], [35]–[46]. Two groups consisting of four EIAV-naïve ponies were inoculated I.V. with 10^3^ TCID_50_ of either EV_NTerm_ or EV_CTerm_. Inoculated ponies were monitored daily for clinical signs of EIA (fever, lethargy, petechiation, diarrhea), and blood samples were taken at regular intervals for measurement of platelets and plasma virus levels (Figure 2). Three of the four EV_NTerm_ ponies rapidly developed acute EIA by three weeks post-infection (Figure 2A–D, Figure 3). Increased temperatures accompanied by drops in platelets, the hallmark of EIA, were observed in all three subjects. Evaluation of plasma viral loads determined that the ponies averaged approximately 10^5^ copies RNA/ml plasma during the infection, and that the febrile episode viremia levels were between 10^6^ and 10^7^ copies RNA/ml plasma. Pony #D47 had three disease episodes during chronic disease and had to be euthanized (Figure 2C). The fourth EV_NTerm_ pony, #G34, had approximately 10-fold lower level of steady state viral RNA and never developed the signs of clinical disease (Figure 2D). Only a single pony from the EV_CTerm_ group developed the clinical signs of EIA (Figure 2E–H, Figure 3). Pony #G33 had plasma viral levels similar to the EV_NTerm_-infected ponies, and experienced clinical EIA accompanied by 10^5^ copies/ml plasma RNA within the first 3 weeks post-infection. The remaining 3 ponies of the EV_CTerm_ group, however, did not experience any clinical symptoms throughout the 90-day observation period (Figure 2E–G, Figure 3). The viral loads of these three animals were also approximately 10-fold lower than their afebrile EV_NTerm_ counterparts, demonstrating fairly steady replication at 10^3^ copies RNA/ml plasma. At the close of the pathogenicity study, 25% of EV_NTerm_-infected ponies lacked clinical signs of disease, while 75% of EV_CTerm_-infected ponies did not develop clinical EIA disease (Figure 3). The low level of virulence observed with the EV_CTerm_ strain made it unsuitable as a pathogenic challenge virus, and hence was not utilized as part of the trial designed to assess protection from disease.

### Clinical, Virological, and Immunological Response to Experimental EIAV_D9_ Vaccination

**Figure 4:**
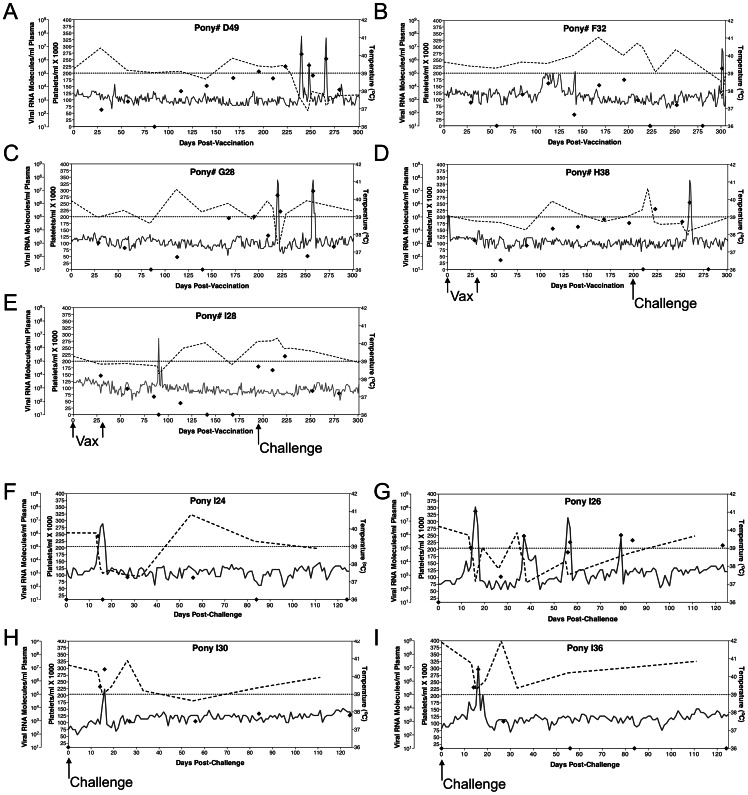
Clinical and virological profiles of EVN_Term_ challenged vaccinated and naïve ponies. The profiles depicted in A–I display the clinical and virological outcomes observed in EIAV_D9_ vaccinated animals (**A–E**), and EIAV naïve animals (**F–I**) upon challenge with 10^3^ TCID_50_ EV_NTerm_ chimeric proviral strain. (**A–E**) Five EIAV-naïve ponies were vaccinated with 10^3^ TCID_50_ EIAV_D9_ I.V. (↑ Vax ↑). Rectal temperature (**–**, right Y axis) and platelet counts (–-, first left Y axis) were followed daily for up to 300 days (X-axis) after the first vaccine dose. Quantification of the virus load (⧫, second left Y axis) was performed on viral RNA extracted from plasma at periodic time points prior to and after virulent virus challenge seven months post-first vaccination with 10^3^ TCID_50_ EV_NTerm_, I.V. (↑Challenge). (**F–I**) Four EIAV-naïve ponies were also challenged with 10^3^ TCID_50_ EV_NTerm_, I.V. (↑Challenge). Febrile episodes were defined by a achieving a combination of two-three features such as: rectal temperature above 39°C in conjunction with thrombocytopenia (platelet decrease of ≥70,000/µl of whole blood), EIAV viral load ≥10^5^ as well as other clinical signs of EIA.

To examine whether protection could be conferred to the EV13 challenge strain by replacing the EV13 gp90 N-terminus with the gp90 N-terminus of the EV0 strain, five EIAV-naïve ponies were vaccinated with our EIAV_D9_ attenuated proviral vaccine strain. Vaccinations, as previously documented [24]–[28], consisted of two inoculations of 10^3^ TCID_50_ of EIAV_D9_ administered at a 1-month interval (Figure 4 A–E). All subjects were monitored daily for clinical signs of adverse reaction to the vaccine or the development of vaccine-associated EIA. Blood samples were drawn at regular intervals for measurement of platelets, plasma virus levels, and EIAV-specific humoral and cellular immune responses. Attenuated vaccine viral loads appeared typical as compared to previously published data, starting at approximately 10^3^ copies and steadily increasing to 10^4^–10^5^ copies, and averaging a steady state level of approximately 10^4^ copies RNA/ml plasma. The average viral load for all vaccinates on the day of challenge was 6×10^4^ copies of RNA/ml plasma. One vaccinate, pony #I28, experienced an increase in rectal temperature pre-challenge, but the mild decrease in platelets, and the lack of viremia during this episode indicate that the fever was non-EIAV related.

**Figure 5:**
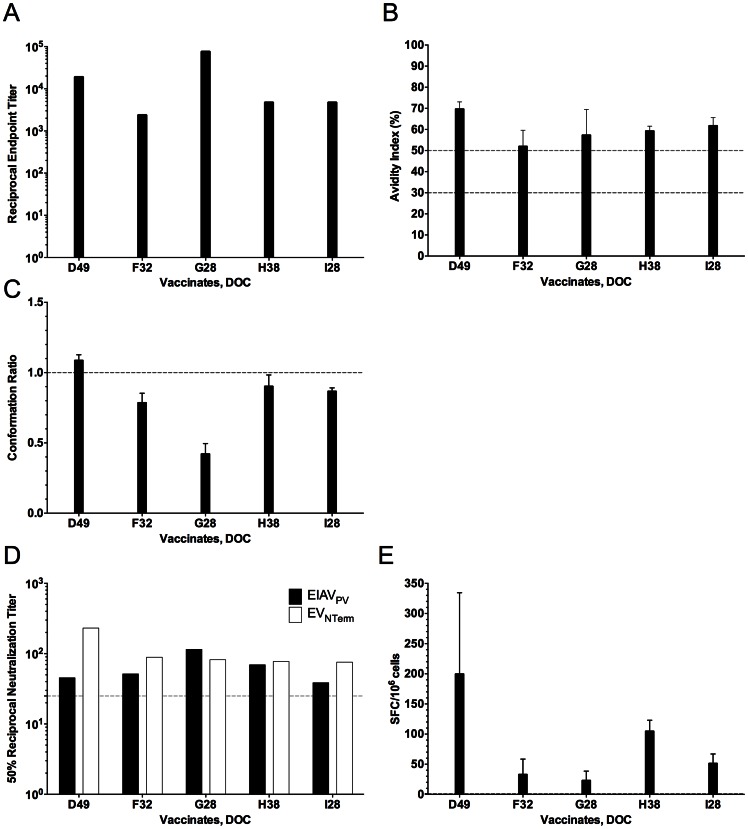
Day of challenge humoral and cellular immune responses of EIAV_D9_ vaccinates. Characterization of the quantitative and qualitative properties of induced EIAV envelope-specific humoral and cellular responses on the day of challenge were conducted in ConA serological ELISA assays of serum antibody (**A**) endpoint titer, (**B**) avidity, and (**C**) conformational dependence; (**D**) 50% serum neutralization titer determinations, and (**E**) INF-γ ELISpot of PBMC, all as described in Materials and Methods. (**A**) Mean serum antibody titers are presented as the log_10_ of the highest reciprocal dilution yielding reactivity two standard deviations above background. (**B**) Mean avidity index measurements are presented as percentages of the antibody-antigen complexes resistant to disruption with 8 M urea. (**C**) Mean conformation dependence values are calculated as the ratio of serum antibody reactivity with native envelope compared to denatured envelope antigen. Conformation ratios greater than 1.0 indicate predominant antibody specificity for conformational determinants, while ratios less than 1.0 indicate predominant antibody specificity for linear envelope determinants. (**D**) The mean reciprocal dilutions of serum from vaccinated horses which neutralized 50% of input EIAV_PV_ or EV_NTerm_, as measured in an infectious center assay. The line (–-) denotes the cut off (≥25) value for valid 50% neutralization titers. (**E**) EIAV Env-specific cellular activity measured as the mean INF-γ ELISpot analysis of EIAV gp90 peptide stimulation of PBMC from vaccinated horses. SFC, Spot-forming Cells.

Day of challenge immune responses in all vaccinates were examined utilizing standard procedures established in multiple pathogenesis and vaccine trials by our research group [15], [18], [19], [23]–[27], [41], [42], [46]. Initial immunogenicity of the vaccine strain was confirmed by reactivity testing in typical USDA-approved commercial diagnostic assays for EIAV infection, the USDA reference agar gel immunodiffusion (AGID) test [47] and the ELISA-based ViraCHEK® assay [24], based on detecting antibody to the viral capsid protein, p26. All experimentally vaccinated horses were seropositive on the day of challenge by these standard assays (data not shown). Quantitative and qualitative humoral and cellular evaluations for each vaccinate were performed next (Figure 5). Concanavalin A (ConA) ELISAs were utilized to characterize the DOC EIAV Env-specific immune responses of the EIAV_D9_ vaccinated ponies. No remarkable differences in the endpoint titer of EIAV Env-specific IgG were observed between vaccinates (Figure 5A). All animals developed a pre-challenge, steady-state reciprocal titer of Env-specific antibodies characteristic of a mature immune response to EIAV, ranging between 10^3^ and 10^4^. The qualitative serological assay of antibody avidity also demonstrated similar envelope-specific antibody responses among the different vaccinates. Steady-state avidity values of 50–70%, indicative of a mature, protective antibody response were observed (Figure 5B). Similarly, DOC antibody conformation ratios demonstrated values indicative of a mature antibody response signaling a switch of antibody specificity from predominantly linear to conformational epitopes, ranging between 0.5 and 1.0 (Figure 5C). Neutralizing antibody titers examined the ability of DOC immune sera to inactivate both the virulent, historical reference EIAV_PV_ strain and the EV_NTerm_ challenge strain infectivity, as summarized in Figure 5D. These data demonstrated that the EIAV_D9_ attenuated candidate generated 50% antibody neutralizing titers around 10^2^, which is typical for the EIAV_D9_ attenuated strain. The neutralizing antibodies of all vaccinates inactivated both EIAV_PV_ and EV_NTerm_ at levels above background, but did not distinguish between the protected from the unprotected vaccinates. Finally, to examine the ability of the EIAV_D9_ vaccine to elicit virus-specific cellular responses, Env-specific reactivity of isolated DOC PBMC were measured in our peptide interferon gamma (IFNγ) ELISpot assay (Materials and Methods) as summarized in Figure 5E. In general, vaccinated ponies demonstrated equivalent Env-specific reactivity, responses averaging between 50–75 SFC/million cells. Overall there were no discriminating differences in humoral and cellular immune responses in the five EIAV_D9_ vaccinated animals.

### Clinical and Virological Profiles of Vaccinated Animals Challenged with EV_NTerm_

Protective efficacy of the immune responses elicited by the attenuated virus inoculation, and the potential of the EV0 N-terminal gp90 sequences to confer protective efficacy to the EV13 backbone, were examined by challenging the immunized ponies with the pathogenic EIAV chimeric strain, EV_NTerm_. Specifically, six months following the second vaccine dose, the five vaccinated ponies and a control group of four EIAV-naïve ponies were challenged with a single dose of 10^3^ TCID_50_ EV_NTerm_. The ponies were monitored daily for clinical symptoms of EIA, and blood samples were drawn at regular intervals (weekly, daily if febrile) for assays of platelets, viral replication, and virus-specific immune responses. The ponies were observed for approximately 120 days post-challenge, which was the equivalent of nearly 320 days of observation for the entire study, at which time they were euthanized.

**Figure 6:**
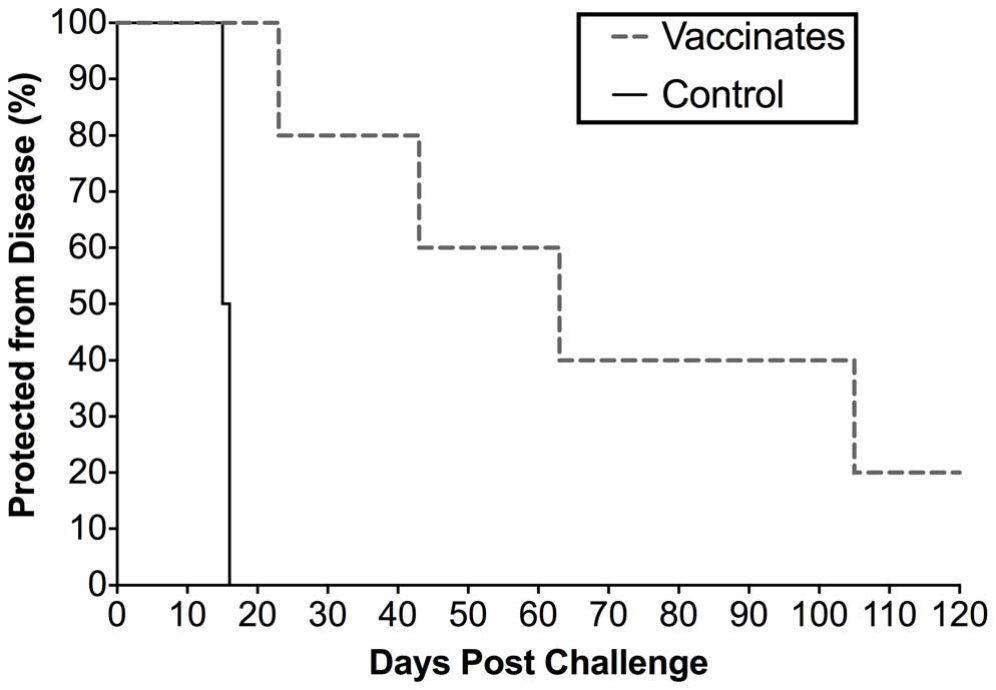
EVN_Term_ chimeric viral strain clinical disease is significantly different than disease in unvaccinated controls. The percentage of EIAV_D9_ vaccinated and unvaccinated control animals that were protected from clinical EIA upon challenge with EV_NTerm_ were plotted as a function of days post-infection. Kaplan-Meier survival plots were generated plotting protection from disease as “0” at the end of the study (120 days post challenge) and disease as “1” on the first day of disease in GraphPad Prism 6.0a (GraphPad Software Inc., LaJolla, Ca.). Gehan-Breslow-Wilcoxon test for statistical relevance demonstrated a significant difference in the survival curves, *P* = 0.0054.

**Figure 7:**
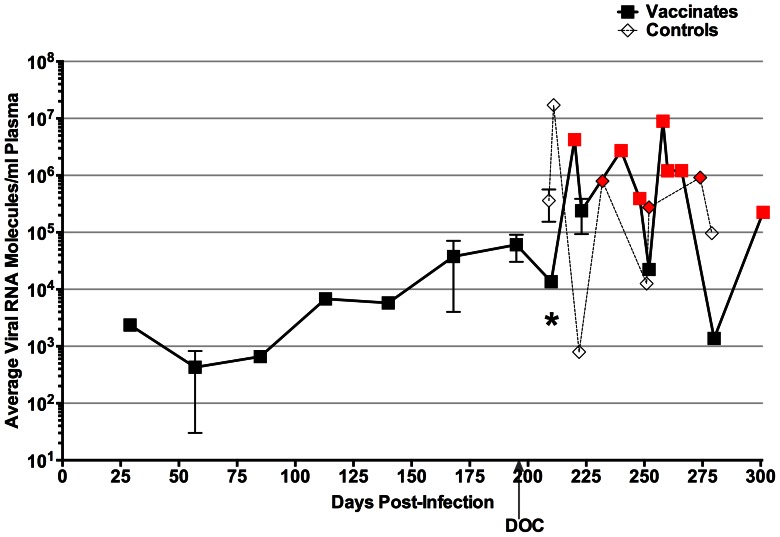
Viral loads in vaccinated ponies and naïve ponies present distinguishing differences. The longitudinal average of the viral loads, quantitated from plasma RNA, for the challenged vaccinates as well as the challenged unvaccinated ponies were plotted as a function of the observation period (approximately 300 days). Red symbols in both groups indicate a single animal’s viral level during febrile episode. DOC, Day of challenge; *, Mann-Whitney statistical results, *P* = 0.015.

As summarized in Figure 4 F–I and Figure 6, all four unvaccinated control ponies succumbed to EIA disease by three weeks post-challenge with EV_NTerm_. Increased rectal temperatures with concurrent incidents of thrombocytopenia were observed by 16 days post-challenge (DPC) in all four animals. Disease-associated plasma viral loads in the control group peaked between 10^6^ and 10^7^ copies of RNA in all four control subjects (Figure 4 F–I). Four of the five vaccinated ponies experienced clinical EIA during the post-challenge observation period (Figure 4 A–E, Figure 6). One EIAV_D9_ vaccinate, #G28, had a fever (23 DPC) within the expected acute stage (28 DPC) while all other vaccinates experienced delayed-onset of disease (between 43 and 105 DPC) or no apparent disease in the case of pony #I28 (Figure 4 A–E, Figure 6). Post-challenge viral loads were higher in the unvaccinated group as compared to the EIAV_D9_ vaccinated animals. Prior to EV_NTerm_-associated acute disease, at 14 DPC, the average viral load in the control group was over 10-fold higher (4×10^5^ vs. 1×10^4^) than that of the EIAV_D9_ vaccinates (*P* = 0.0159, Figure 7). Disease-associated viral loads in the unvaccinated group also trended not quite 10-fold higher than those in the vaccinated animals at acute disease averaging approximately 10^7^ copies in the former and 10^6^ copies RNA/ml plasma in the latter (Figure 4, 7). Kaplan-Meier survival curves of the protection from disease data demonstrate a statistically significant difference between unvaccinated control animals and the EIAV_D9_ vaccinates (Figure 6). Hence, the N-terminus of the EV0 gp90 region did not achieve completely the high levels of protection observed previously with EIAV_D9_ vaccination and challenge with EV0. However, EV_NTerm_ disease in the EIAV_D9_ vaccinated animals was significantly delayed (*P* = 0.0054) as compared to unvaccinated ponies with statistically significantly lower levels of viral levels (*P* = 0.015) prior to the onset of acute disease.

## Discussion

Among the diverse AIDS vaccine strategies tested to date in animal lentivirus models, attenuated lentivirus vaccines have uniformly provided the highest levels of immunogenicity and protection from disease [24], [30], [48]–[58]. Important lessons on the fundamentals of vaccine immunogen design, development, trial approaches, and correlates of protection can be learned from studies of this highly successful vaccine regimen. For approximately 15 years we have utilized our EIAV attenuated vaccine platform to inform thoughtful immunogen composition of vaccines for lentivirus diseases. We recently published a report that established an absolute relationship between defined natural lentivirus Env variation and vaccine efficacy. The study presented here was designed to evaluate if the vaccine efficacy associated with the high level of observed protection to the EV0 strain (100%) could be conferred to the EV13 strain (40% protection) through selected mapping of EV0 gp90 amino acid sequences.

While the generation of chimeric strains is simple in concept, it is not always simple in practice. Both the parental strain EV0 and EV13 Env gp90s are from viral isolates that arose from the same experimental infection [19], [42], [44] during the acute and inapparent stages of disease (EV13 evolving approximately 1200 days after EV0) and were therefore “siblings” of the same biological clone, EIAV_PV_. This ancestral relationship between the strains precipitated the anticipation of a simple swap between the N-terminal and the C-terminal halves of the respective isolate Env gp90s with little expectation of complications. Cloning occurred readily, and all *in vitro* evidence (Figure 1B) indicated the strains had very similar replication characteristics. *In vivo* infection studies with the two strains yielded more complicated results.

The EV_NTerm_ induced disease in three of four infected ponies, achieving the somewhat expected level of virulence. The lack of virulent disease in 75% of the EV_CTerm_ animals (Figure 2, 3) was far more unexpected. The cause of the observed lower viral replication, which played a role in the lack of clinical disease, was not clear. *In vivo* trials of the EV13 clone demonstrated a higher level of virulence than the EV0 in pathogenicity studies prior its use as a challenge strain (data not shown, [27]). The EV_CTerm_ contains the N-terminus of the gp90 of the EV13 clone, which contains the highly variable V3–V4 genomic region that encodes the principal neutralizing domain (PND). Furthermore, previous studies by us and others determined that the V3–V4 region of gp90 is an area highly associated with neutralization sensitivity or resistance and is therefore thought to be a determinant of viral virulence [18], [42], [59]–[62]. Thus, having the EV13 V3–V4 region in the EV_CTerm_ chimeric clone lead to the understandable, yet inaccurate, theory that the chimeric provirus would be highly virulent. However, it is likely that amino acid differences in the reciprocal termini of the parental proviral clones compensate for structural differences that are not accounted for in the chimeric exchanges. The gp90 conformation is therefore potentially compromised just enough that simple *in vitro* replication appears normal yet the complications of *in vivo* replication yield a moderately attenuated strain. Similar to this finding, a natural EIAV isolate with a 14 amino acid deletion in the PND region of the V3 was isolated, cloned, and evaluated for replication *in vitro* and *in vivo*. The isolate, which was also predicted to be highly pathogenic, did not cause disease in animals through a yearlong observation period [18]. Taken together with the results of the current pathogenicity trial, the data suggest limitations of predicting *in vivo* viral virulence based on *in vitro* determinations of neutralization resistance and sensitivity. More important towards immunogen development are considerations of conformational differences between strains and their respective effects on the recognition of immune response that do not necessarily include standard measures of 50% neutralization titers. Unfortunately, since EIAV lacks a crystal structure for its gp90 protein, reasonable estimates of differences in protein structure are not possible here. The outcome of the pathogenicity trial precluded the use of the EV_CTerm_ as a challenge strain for assessment of protection from disease. While the reciprocal gp90 exchange in the EV_CTerm_ strain would have been ideal, the lack of virulence in the EV_CTerm_ strain would have made challenge results impossible to interpret. Therefore, the EIAV_D9_ vaccine trial commenced with a single chimeric challenge strain.

EIAV_D9_ vaccination yielded a statistically significantly lower level of post-challenge EV_NTerm_ viral levels and delay in clinical EIA disease (Figure 6, 7). This delay in disease was also a longer delay than the typical time to disease noted in challenge studies with the parental EV13 [27]. However, four of five animals still developed clinical EIA, unlike the high levels of protection observed with EV0 parental strain in the previous study. Based on the virulence and neutralization sensitivity associated with the V3–V4 epitopes, it was expected that the chimeric EV_NTerm_ strain (EV0 N-terminus), which is homologous in the N-terminal region to the EIAV_D9_ vaccine, would have increased protective efficacy compared to the EV13 parental strain. Moreover, the assumption that protective antibodies would be generated to the PND region by the vaccine strain, in light of the 100% homology between the EIAV_D9_ and EV_NTerm_ in the V3–V4 region, prejudiced expectations of higher levels of protection. Neither protective nor virulent Env epitopes have been mapped to the C-terminus of the gp90 protein. An earlier report did note a neutralization domain in the V5 region [63], but subsequent studies on regional epitope conferment of neutralization sensitivity and resistance yielded no indications of consequence in the C-terminus. The most notable change within the 26 amino acid differences between the EIAV_D9_ C-terminus and the EV13/EV_NTerm_ C-terminus was the reduction in the number of potential N-linked glycosylation sites (four fewer sites, Figure 1A). This reduction in glycosylation typically would be predicted to render the region more vulnerable to neutralization and hence, less pathogenic. It is possible that sensitivity to neutralization, with a less dense glycosylation shield, is what allowed for delay in disease. In light of previous findings of immune escape and the evolution of EIAV with each febrile episode [19], [42], [44], it is highly likely that the isolates that escaped vaccine immunity evolved from the infectious inoculum given the amount of time between challenge and disease in many of the vaccinates. Recognizing the effects that glycosylation has on protein structure [64], [65], it is also very probable that the differences in glycosylation cause subtle differences in the EV_NTerm_ structural conformation of the gp90 protein which allows escape of the EIAV_D9_–induced protective immunity.

Examination of the day of challenge immune response did not reveal any apparent correlates of protection. Env-specific serological analyses were indicative of a mature protective immune response as demonstrated previously [25]–[27]. The IFNγ ELISpot assay of cellular reactivity is new to our studies and has no historical reference, but results while indicative of Env-specific responses did not distinguish between the single protected vaccinate and the other four unprotected vaccinates. One point that could be made regarding the Env-specific serological results was the moderately low conformation ratio for pony #G28. Most vaccinated animals display DOC antibody conformation ratios higher than 0.5 and are closer to 1.0, indicative of an increase in recognition of predominantly conformational epitopes instead of linear epitopes. This relatively low antibody conformation dependence may potentially explain why pony #G28 is the only vaccinate to break with acute disease within the normal timeframe. All vaccinated animals had neutralization titers to EIAV_PV_ and EV_NTerm_. The 50% neutralizing antibody titers were typical of EIAV-specific immunity but did not distinguish between the protected vaccinate and the other four unprotected vaccinates. While antibody responses clearly play a key role in EIAV vaccine efficacy, results presented here support a platform that adopts refocusing of assays to find correlates of key, non-neutralizing antibody measures, as suggested by the findings of the well reported HIV RV144 vaccine trial. Specifically the finding of V1–V2 binding antibodies, not neutralizing antibodies, as a correlate of protection share a similar theme with the findings observed in the current trial [66]–[68].

The data presented in this report demonstrate some interesting observations of *in vitro* mapping of *in vivo* interactions with lentiviral proteins. The pathogenicity trial of the chimeric EIAV strains demonstrated that reciprocal swaps between variant Env species of virulent isolates do not always yield a virulent virus. Furthermore, the current observations indicate that *in vitro* assays of regional epitope determinants of neutralization sensitivity and resistance do not necessarily reveal *in vivo* epitopes of virulence. Vaccine efficacy studies demonstrated that homology between the N-terminus of the vaccine and challenge strain gp90s was capable of inducing immunity that resulted in significantly lower levels of post-challenge virus and significantly delayed the onset of EIA disease. However, a homologous N-terminal region alone could not impart complete protection. Both the pathogenicity and vaccine efficacy studies suggest that large regional residue exchanges potentially affect conformation by excluding compensatory mutations. Smaller sequence swaps are less likely to have large-scale conformational effects that could alter the virulence of the strains. These studies ultimately emphasize the importance of focusing vaccine efforts on structural accuracy and conformational integrity of Env immunogens. Furthermore, future studies should aim on engineering new assays of antibody binding that distinguish correlates of protection that inform development of novel Env immunogens that elicit a broader and more effective recognition of variant Env species.

## Materials and Methods

### Design, Construction, and Production of Chimeric EIAV virus Strains

Chimeric EIAV proviral strains were produced from the viral challenge strains developed for a previous study on variant envelope effects on vaccine efficacy [27], [28]. Two chimeric proviral EIAV strains were created utilizing the virulent EV0 and EV13 EIAV strains [27] (gp90 envelope sequences: GenBank accession numbers AF016316.1, and AY858747.1, respectively). Proviral strains EV_NTerm_ and EV_CTerm_ (Figure 1) were generated utilizing a combination of PCR-generated fragments and standard restriction endonuclease digestions to create chimeric proviral strains from the N-terminus and C-terminus of both EV0 and EV13, splitting and recombining the strains between the fourth and fifth variable regions (Figure 1). Standard PCR conditions were employed [18], [27]. The resultant PCR products of the desired envelope fragments were gel purified, digested, and cloned back into the backbone of the respective counterpart EIAV strains [27]. All proviral clones were sequenced to verify the swapped envelope sequences. Sequencing reactions were performed with the Taq Dye Deoxy Terminator Cycle Sequencer Kit (Applied Biosystems, Foster City, CA) using internal EIAV primers [19], [42], [44]. DNA sequences were resolved with an ABI Prism 373 DNA sequencer (Applied Biosystems, Foster City, CA). Viral stocks were prepared by harvesting the supernatant medium from equine dermal (ED) cells (ATCC CRL 6288) as preciously described [69]. Viral stock titers were determined utilizing our infectious center assay (cell-based ELISA) in FEK cells, described previously [70], [71]. *In vitro* viral replication kinetics of the chimeric strains was determined as previously described by reverse transcriptase (RT) activity analysis of supernatants from infected FEK cells [43], [69], [72].

### Experimental Subjects, Inoculations, Clinical Evaluation, and Sample Collection

All equine procedures were conducted in accordance with the recommendations in the Guide for the Care and Use of Laboratory Animals of the National Institutes of Health at the Gluck Equine Research Center of the University of Kentucky according to protocols approved by the University of Kentucky IACUC (#01058A2006). The animals were monitored daily and maintained as described previously [25]–[27], [40], [42]. Platelet numbers were determined using the IDEXX VetAutoread Hematology Analyzer (IDEXX Laboratories Inc., Westbrook ME). Clinical EIA (fever) episodes were determined on the basis of rectal temperature and platelet count (rectal temperature >39°C; platelet number <100,000/µl of whole blood) in combination with the viremic presence of infectious plasma virus (>10^5^) [13], [16], [40], [42], [73]. Samples of whole blood, serum, and plasma were collected weekly as well as daily during fever episodes. Plasma samples were stored at −80°C until used to determine plasma viral RNA level. Serum samples for serological analysis were stored at −20°C. Peripheral blood mononuclear cells (PBMC) were isolated using Ficol-Paque Plus™ (Amersham Biosciences, Piscataway, NJ) gradient centrifugation. PBMC were cryopreserved and stored in liquid nitrogen for ELISpot analysis of INFγ production. During the course of these experiments ponies that demonstrated severe disease-associated symptoms resulting in distress as outlined by the University of Kentucky IACUC were euthanized.

#### Experimental infections to determine virulence

Eight outbred, mixed-breed ponies were separated into two groups of four and experimentally inoculated intravenously with 10^3^ TCID_50_ of either chimeric strain EV_NTerm_ and EV_CTerm_. Rectal temperatures and clinical status were recorded daily. The ponies were monitored for clinical, virological, and immune responses as described above.

#### Attenuated vaccine inoculations and challenge

Eight outbred, mixed-breed ponies of mixed age and gender and serognegative for EIAV were utilized. Daily rectal temperatures and clinical status were recorded. The EIAV_D9_ attenuated virus stock was produced and vaccinations performed as described [25]–[27]. Five ponies were vaccinated and four were maintained as EIAV-naïve, unvaccinated control animals. All vaccinated ponies received two inoculations of EIAV_D9_ at 1-month intervals by intravenous injection of 10^3^ TCID_50_ as previously described [26], [27]. Vaccinated ponies were challenged six months post-second inoculation with 10^3^ TCID_50_ EV_NTerm_. Unvaccinated controls were challenged at the same time as their vaccinated counterparts. Ponies were monitored daily for clinical symptoms of EIA. Virological and immunological responses were monitored as described above and in the following sections.

### Viral RNA Purification and Quantitation

Circulating plasma viral load analysis of all animals was analyzed using a previously described Taqman quantitative real-time multiplex RT-PCR assay based on *gag*-specific amplification primers [74]. The standard RNA curve of the assay was linear within the range of 10^1^ molecules as a lower limit and 10^8^ molecules as an upper limit. Statistical significance of the differences in the average viral loads in challenged vaccinated animals versus naïve animals was determined using a nonparametric, Mann-Whitney test (Prism 6.0a, GraphPad Software, Inc., La Jolla, CA).

### Quantitative and Qualitative Serological Analyses

Detection of serum antibody reactivity to the EIAV capsid protein p26 was conducted using the ViraCHEK®/EIA kit per the manufacturer’s instructions (Synbiotics Laboratory, Via Frontera, San Diego, CA). Serum samples were also evaluated for seroreactivity by the standard agar gel immunodiffusion (AGID) procedure [47] diagnostic assay for EIA. Serum IgG antibody reactivity to EIAV envelope glycoproteins was assayed quantitatively (end point titer) and qualitatively (avidity index, conformation ratio) using our standard ConA ELISA procedures as described previously [15], [41], [75]. Virus neutralizing activity to EIAV_PV_ (historical reference and homologous to vaccine Env) as well as the EV_NTerm_ challenge virus strain mediated by immune sera was assessed in an indirect cell-ELISA based infectious center assay using a constant amount of infectious virus (50 units) and sequential 2-fold dilutions of serum [41], [71]. All serological analyses experiments were performed in triplicate.

### INFγ ELISpot Analysis of PBMC

MultiScreenHTS-IP Filter plates (Millipore, Bedford, MA) were coated with 7.5 µg/ml monoclonal mouse-anti bovine IFNγ antibody at room temperature for four hours. Plates were washed with PBS and blocked for one hour with RPMI media (2.5% fetal equine serum, 2 mM glutamine, 100 U/ml penicillin/streptomycin, and 55 µM 2-mercaptoethanol). Cryopreserved PBMC were thawed and 2×10^5^ cells added to triplicate wells. Env gp90 peptides [27], [33], [34] were added at a final concentration of 20 µg/ml. Medium only (negative) controls did not contain peptides. Phytohemaglutinin (PHA, 10 µg/ml) was used as positive control. Plates were incubated at 37°C/5% CO_2_ for 16 hours. Plates were washed with PBS and biotinylated mouse-anti bovine IFNγ antibody (diluted in PBS/0.5% FBS) added to each well at the final concentration of 0.25 µg/ml. After incubation for two hours at 37°C, plates were washed with PBS. Streptavidin-alkaline phosphatase (Mabtech, Mariemont, OH) was added and the plates incubated for one hour. Spots were developed by incubating the plates with substrate BCIP-NBTPLUS (Mabtech, Mariemont, OH) for 30 min and stopped by rinsing with distilled water. Spots were scanned and analyzed on an Immunospot Analyzer (Cellular Technology, Cleveland, OH). The number of IFNγ producing cells were calculated as mean values of triplicate wells, minus background medium controls, and shown as spot forming cells (SFC per million PBMC).
